# Conformation-selective rather than avidity-based binding to tumor associated antigen derived peptide-MHC enables targeting of WT1-pMHC low expressing cancer cells by anti-WT1-pMHC/CD3 T cell engagers

**DOI:** 10.3389/fimmu.2023.1275304

**Published:** 2023-11-10

**Authors:** Even Walseng, Bo Wang, Chunning Yang, Pooja Patel, Chihao Zhao, Hanzhi Zhang, Peng Zhao, Yariv Mazor

**Affiliations:** Biologics Engineering, Biopharmaceutical R&D, AstraZeneca, Gaithersburg, MD, United States

**Keywords:** T cell engager, DuetMab, bispecific, conformation, avidity, TCR mimic, target density

## Abstract

T cell engagers, a category of T cell-retargeting immunotherapy, are rapidly transforming clinical cancer care. However, the lack of tumor-specific targets poses a significant roadblock for broad adaptation of this therapeutic modality in many indications, often resulting in systemic on-target off-tumor toxicity. Though various tumor-derived intracellular mutations provide a massive pool of potential tumor-specific antigens, targeting them is extremely challenging, partly due to the low copy number of tumor associated antigen (TAA)-derived pMHC on tumor cell surface. Further, the interplay of binding geometry and format valency in relation to the capacity of a T cell engager to efficiently target low density cell-surface pMHC is not well understood. Using the Wilms’ tumor 1 (WT1) oncoprotein as a proof-of-principle TAA, combined with an array of IgG-like T cell engager modalities that differ in their anti-TAA valency and binding geometry, we show that the ability to induce an immunological synapse formation, resulting in potent killing of WT1 positive cancer cell lines is primarily dependent on the distinct geometrical conformations between the Fab arms of anti-WT1-HLA-A*02:01 and anti-CD3. The augmented avidity conferred by the binding of two anti-WT1-HLA-A*02:01 Fab arms has only minimal influence on cell killing potency. These findings demonstrate the need for careful examination of key design parameters for the development of next-generation T cell engagers targeting low density TAA-pMHCs on tumor cells.

## Introduction

T cell-redirecting therapies, including chimeric antigen receptor (CAR) T cells and T cell engagers, have transformed the therapeutic landscape of clinical oncology (1–5). By reprogramming endogenous T cells with tumor associated antigen (TAA)-specific CAR-T or co-engaging T cells and target cells via CD3/TAA-targeting bispecific antibodies, the native pMHC specificities of the polyclonal repertoire of patients’ T cells can be overridden to retarget tumors (5). Multiple clinical trials using CD19 targeting CAR-T cells and T cell engagers (TCEs) have demonstrated robust efficacy and durable responses for treatment of hematological malignancies (6–9). However, their efficacy in the treatment of solid tumors has yet to be proven. In comparison with CAR-T cells that require patient-specific manufacturing, TCEs have the potential to provide the ease of off-the-shelf therapeutic administration (5).

Successful application of TCEs against solid tumors is complicated by a number of challenges present in the tumor microenvironment (TME), including the general exclusion of infiltrating cytotoxic lymphocytes and the abundance of immunosuppressive immune and non-immune cells. Furthermore, poor differential expression of TAAs on tumors versus normal tissues (10–13) makes TAA selection a critical decision that ultimately influences the fate of a candidate drug. As most TAAs are not tumor-specific, targeting these antigens with potent T cell-redirecting therapeutics carries the risk of on-target off-tumor cell killing, which may cause severe toxicity to normal tissues (14–17). Thus, careful selection of TAAs for T cell engagers is a prerequisite for maximizing therapeutic index and minimizing the risk for adverse effects.

Conversely to TAAs in the form of cell surface receptors, many tumors harbor a large number of mutations in their intracellular proteome, providing an abundant pool of potential tumor-specific target antigens. Targeting of intracellular oncoproteins using biological drug modalities have been a long-sought-after goal for effective cancer treatment (18–20). Though long deemed “undruggable”, recent advances in protein engineering and CMC manufacturing have advanced the capabilities of targeting these tumor-specific intracellular proteins (21–23).

Targeting of intracellular oncoproteins can be achieved by utilizing TCR mimic mAbs (TCRm) (24–27). These antibodies recognize linear peptide epitopes presented in the context of cell-surface MHC molecules in a manner similar to that of T cell receptors (TCR). Deployment of TCRm as a targeting arm of a TCE can facilitate the targeting of intracellular proteins via redirection of T cells. While TCRm TCEs in the form of scFv bispecific T-cell engager (BiTE) modalities were previously reported, the interplay of antibody binding geometry and format valency in relation to the capacity of a TCRm TCE to effectively target and eradicate tumor cells displaying low density pMHC has not yet been interrogated (25, 26, 28).

We have previously developed a modular platform, named DuetMab, for flexible and efficient bispecific antibody generation (29). Making use of this platform, we uncovered novel insights into how the intrinsic affinity and avidity of the Fab arm affect target selectivity (29–32).

In this study, we report the design, optimization and characterization of a DuetMab based CD3 T cell engager, named T cell engager DuetMab (TED), which can target tumor cells expressing a low copy number of TAA-derived pMHCs. By using the WT1 oncogene derived RMFPNAPYL peptide presented on HLA-A*02:01 as proof of concept for a low density target, we found the geometric proximity between the anti-TAA Fab arm and the anti-CD3 arm, rather than the added avidity resulting from the two anti-TAA Fab arms, to be the driving force for more efficient immunological synapse (IS) formation.

Our results provide important insights into the critical role geometry plays when targeting low density receptors such as pMHCs derived from tumor-specific mutations. These design parameters should be carefully considered when developing next-generation TCEs with enhanced potency and improved therapeutic index.

## Materials and methods

### TED construct generation and production

WT1 and EGFR TEDs were constructed by cloning V_H_ and V_L_ of anti-WT1-HLA-A*02:01 (clone ESK1), anti-EGFR (clone GA201), and anti-CD3 (clone UCHT1) antibodies into pDuet-Heavy and pDuet-Light vectors, respectively (29, 33–35). After sequence verification, the pair of vectors encoding each TED was transiently transfected into CHO-K1 cells at 1:1 ratio, and the supernatant was harvested 10 days after transfection. The supernatant was sterile-filtered and loaded on a 5ml MabSelect SuRe column (GE Healthcare). The bound antibodies were washed and eluted. After buffer exchange in phosphate buffered saline (PBS), pH 7.2 (Life Technologies), they were loaded on a Superdex 200 16/600 column (GE Healthcare). After pooling the monomer fractions, the antibodies were concentrated using Amicon Ultra-15 Centrifugal Filter Units with 50 kD NMWL (Millpore Sigma). The purity, oligomeric state and endotoxin contamination of the purified antibodies were determined by Bioanalyzer A (Agilent), analytical size exclusion chromatography, and Endosafe LAL test cartridges (Charles River) to ensure they have >95% correct pairing, >98% monomer, and <1EU/mg endotoxin. After these quality control measures, the antibodies were flash frozen in liquid nitrogen and stored at -80°C.

### Blood cell isolation

Peripheral blood mononuclear cells (PBMC) were fractionated from the blood of healthy donors collected in BD Vacutainer CPT Mononuclear Cell Preparation Tube with sodium heparin (BD) following the manufacturer’s instruction. After fractionation, the PBMCs were further processed with human pan T cell isolation kit (Miltenyi Biotec) or EasySep human T cell isolation kit (STEMCELL Technologies) for untouched T cell isolation.

### Tumor cell lines and primary AML cells

OVCAR3 and U266B1 cells were maintained in RPMI 1640 medium (Gibco) supplemented with 20% and 15% HI-FBS (Gibco), respectively. K562 cells were cultured in IMDM (Gibco) supplemented with 10% HI-FBS. All cell lines were obtained from American Type Culture Collection.

Primary AML cells were purchased as frozen cells from Discovery Life Sciences and used directly in the assay upon thawing and resting overnight.

### Cytotoxicity and T cell activation assay

Target tumor cells were labelled with CellTrace™ Violet (Life Technologies) and mixed with primary T cells at a ratio of 1:5. The cell mixture containing 60,000 total cells were dispensed into 96-well U-bottom tissue-culture treated plate (Corning), and the corresponding TEDs were 4-fold serially diluted starting from 1μg/ml and added into the coculture. After 24h incubation at 37°C, the cells were spun down by centrifugation at 300g for 2min, and the supernatant was transferred to a new 96-well plate and stored at -80°C. The cells were then lifted with Accutase (Life Technologies), and washed with FACS buffer composed of PBS, pH 7.2, 2% HI-FBS, 2mM EDTA, and 0.01% sodium azide. The cells were stained with a master mix made of propidium iodine (PI, Life Technologies), anti-CD4-FITC (clone RPA-T4, Biolegend), anti-CD8-PE-Cy7 (clone RPA-T8, Biolegend), anti-CD25-PE (clone M-A251, Biolegend), and anti-CD69-APC (clone FN50, Biolegend) in FACS buffer, washed, and acquired on a FACSymphony™ (BD). FlowJo v10.9 was used to analyze the data (BD). Cytotoxicity was calculated as (number of live target cells without TED – number of live target cells with TED)/(number of live target cells without TED). T cell activation was calculated as (number of live CD25^+^CD69^+^CD4^+^ (or CD8^+^) cells)/(number of live CD4^+^ (or CD8^+^) cells).

### Cytokine release assay

The inflammatory cytokine concentrations in the coculture supernatant were determined with MSD Multi-Spot 96-well plate customized for detection of IL-2, IFN-γ, TNF-α and IL-6 (Meso Scale Discovery) following the manufacturer’s protocol. Briefly, the plate was first blocked with Blocker B solution and washed. Calibrators was diluted in Diluent 1, and the coculture supernatant was thawed on ice. 25 μl of calibrators and supernatant were then transferred into the plate. After incubation, the detection antibody solution was added. After wash, Read buffer T was added, and the plate was read immediately. Cytokine concentrations were calculated using Discovery Workbench software.

### Octet binding assay

Binding of WT1 TEDs to soluble WT1-HLA-A*02:01 monomer (MBL International) and CD3 (Sino Biological) was measured on an Octet384 (ForteBio). For concurrent binding, CD3δ & CD3ϵ heterodimers were loaded onto Penta-His sensors (ForteBio). TEDs were diluted to 30 μg/ml in PBS, pH 7.2, 3 mg/ml BSA, and 0.05% (v/v) Tween-20 (assay buffer) before use for association with CD3 loaded sensors. After wash, the sensors were dipped into WT1-HLA-A*02:01 monomer solution for association.

For binding of TEDs to WT1-HLA-A*02:01 monomer, biotinylated WT1-HLA-A*02:01 was loaded onto streptavidin sensors. The loaded sensors were subsequently dipped in diluted TEDs and then washed in assay buffer.

Octet 384 software v.7.2 was used for data analysis.

### Mass spectrometry

Briefly, reduced liquid chromatography mass spectrometry (rLCMS) was carried on an Agilent 1200 series HPLC coupled to an Agilent 6520 Accurate-Mass Q-TOF LC/MS with an electrospray ionization source. 2 μg of reduced antibody were loaded onto a Poroshell 300SB-C3 column (2.1 × 75 mm, Agilent) and eluted at a flow rate of 0.4 mL/min using a step gradient of 60% B after 6 min (mobile phase A: 0.1% Formic acid in water and mobile phase B: 0.1% Formic acid in acetonitrile (JT Baker). The data were collected and processed using Agilent Mass Hunter software from Agilent Technologies.

### Structure modeling

The structure model of TCR-CD3- WT1-HLA-A*02:01 complex is generated by the superposition of the TCR extracellular domain of PDB 7PHR (TCR-CD3-HLA) (36) and PDB 6RSY (sTCR- WT1-HLA-A*02:01) (37). The structure model of αCD3(UCHT1) Fab-CD3 by a combination of PDB 1XIW (UCHT1scFv-sCD3) (33) and an AlphaFold model of UCHT1 Fab (38). The structure model of αWT1 (ESK1) Fab- WT1-HLA-A*02:01is obtained from PDB 4WUU (37). The structure model of Fc was obtained from a previous report (39). The max αCD3-αWT1 paratope distance models for WT1 TED, WT1 TED one-arm, WT1 TED2 split, WT1 TED2 tandem, and BiTE are built using the elements from the above model with the theoretical max distances in the loop regions using PyMOL. The models of immunological synapses formed by WT1 TED2 split are built with models mentioned above, using the relative HLA to TCR orientation from the TCR-CD3- WT1-HLA-A*02:01 complex model with linker distances below the theoretical max distance by PyMOL (40–42).

### Statistical analysis

Statistical analyses were performed with Prism 9.5.1.733 (GraphPad). Data sets were analyzed with parametric 2-tailed Student’s t-tests. p values <0.05 were considered significant. The coefficients of determination (R2) were calculated using the Pearson correlation. Trend lines were calculated by simple linear regression.

## Results

### Generation of full-length WT1 TED with two split anti-TAA Fab arms

**Figure 1:**
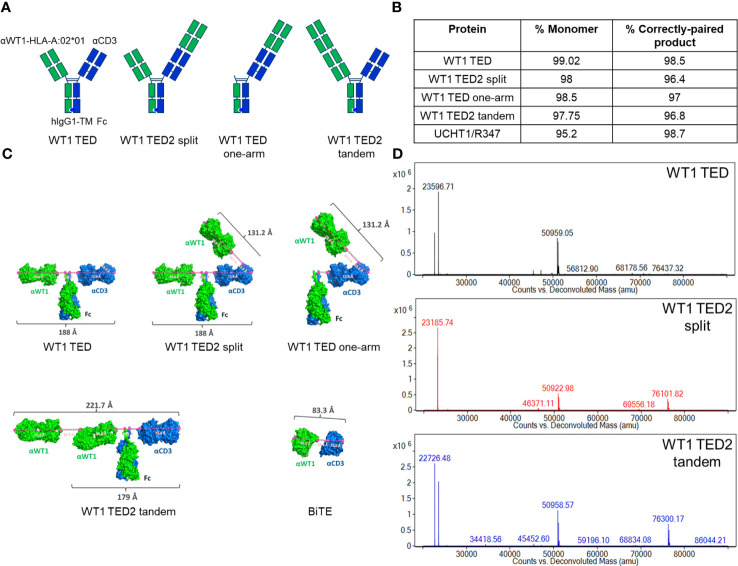
Development of WT1 TEDs. **(A)** Design schematic of WT1 TED, WT1 TED2 split, WT1 TED one-arm and WT1 TED2 tandem. **(B)** Production characteristics of WT1 TED, WT1 TED2 split, WT one arm and WT1 TED2 tandem, with UCHT1-R347 DuetMab as a control. **(C)** Structural models of WT1 TED, WT1 TED2 split, WT1 TED one-arm, WT1 TED2 tandem and BiTE generated in PyMOL to measure the distance between anti-WT1-HLA-A*02:01 and anti-CD3 binding paratopes. **(D)** LC-MS spectra of WT1 TED, WT1 TED2 split and WT1 TED2 tandem.

**Figure 2:**
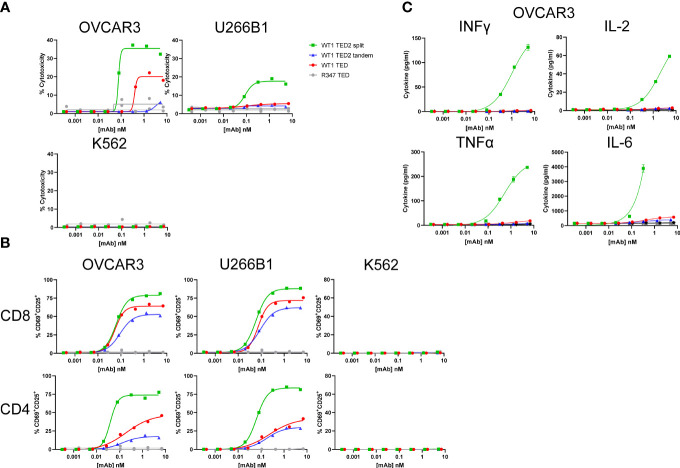
WT1 TED2 split elicits robust cytotoxicity against WT1-HLA-A*02:01^+^ tumor cells. **(A)** Cytotoxicity induced by WT1 TEDs against OVCAR3 and U266B1 and cells, with R347 TED serving as negative control. Primary T cells were incubated with target cells at E:T=5:1 for 24h and target cell cytotoxicity was assessed by FACS. HLA-A*02:01^-^ K562 cells served as a negative control. **(B)** T cell activation induced by WT1 TEDs, evaluated by T cell surface CD69 and CD25 expression. **(C)** Inflammatory cytokine release elicited by WT1 TEDs against OVCAR3 cells. Cytotoxicity and cytokine concentrations were reported as the mean of two replicates, and error bar represents standard error of the mean. T cell activation was reported from one replicate. Representative data using primary T cells from one out of three donors were presented.

We have previously established the DuetMab platform for facile bispecific antibody generation (29, 31, 32, 43, 44). We anticipated that this platform also should be suitable for generation of TCEs targeting different TAAs. For the purpose of this study, we initially designed a DuetMab molecule composed of the variable domains of the anti-WT1-HLA-A*02:01 antibody ESK1-targeting the RMFPNAPYL peptide presented on HLA-A*02:01 with a reported affinity of 0.2 nM as the anti-TAA arm (45–47) and the anti-CD3 antibody UCHT1 with a reported affinity of 1.6 nM as the T cell binding arm (**Figure 1A**) (33, 34). The targeting of this pMHC has been demonstrated to be an effective strategy against multiple malignant indications clinically and was found to be a good candidate for a low density TAA given the very limited amount of relevant pMHC presented on each cell (18, 20, 21, 23, 48–52). To minimize undesired Fc-dependent effector cell and complement recruitment, we used a human IgG1 backbone containing Fc mutations resulting in attenuated effector functions (53). WT1 TED was produced as described for other DuetMabs using standard expression and purification methods (29, 31, 32, 43, 44). WT1 TED displayed comparable expression as its parental antibodies with low level of aggregation (**Figure 1B**). Preliminary functional assessment showed that although WT1 TED elicited T cell activation upon target cell engagement, it induced suboptimal cytotoxic potency (**Figures 2A, B**; **Supplementary Table 2**). To better understand the origin of the suboptimal cytotoxicity, we took advantage of the flexibility that our IgG-like TED format provides to structurally model modalities that differ in their anti-TAA valency and binding geometry - WT1 TED2 split, WT1 TED one-arm and WT1 TED2 tandem. The WT1 TED2 split format has a second anti-WT1-HLA-A*02:01 Fab fused to the N-terminus of the anti-CD3 arm. The WT1 TED one-arm is a monovalent format with an anti-WT1-HLA-A*02:01 WT1 arm fused N-terminal to the CD3, whereas the WT1 TED2 tandem format has a second anti-WT1-HLA-A*02:01 Fab appended to the N-terminus of the original anti-WT1-HLA-A*02:01 arm (**Figure 1A**). When comparing the structural models of these formats with that of anti-WT1-HLA-A*02:01/anti-CD3 BiTE (**Figure 1C**), it became apparent that the distance between the binding paratopes of the TAA and CD3 arm in the current WT1 TED format was suboptimal for IS formation. Compared to the 83.3 Å distance between anti-WT1-HLA-A*02:01 paratope and anti-CD3 paratope in BiTE, the distance between the two paratopes in WT1 TED is 188.0 Å, larger than the reported 140 Å distance between a T cell and a tumor cell deemed as essential for synapse formation and optimal killing of tumor cells (36, 54, 55) (**Supplementary Figure 1**). A table of the measurements are listed (**Supplementary Table 1**). As a result of the lack of other canonical synapse components, the high affinity of each target receptor engaging Fab may not compensate for the short duration of the synapses, as suggested by several studies on the synapses induced by CAR-T cells (48, 49). The extended length between the two arms, exceeding 140 Å, may result in less efficient formation and reduced duration of the immunological synapses between T cells and tumor cells, especially given the low number of WT1-HLA-A*02:01 pMHCs on tumor cell surface (34, 55–57).

In terms of production of the molecules, the addition of a second anti-WT1-HLA-A*02:01 Fab to either arm did not significantly affect the yield, or monomer fraction (**Figure 1B**). The LC-MS spectra of WT1 TED, WT1 TED2 split and WT1 TED2 tandem showed the presence of the heavy and light chains (**Figure 1D**). The choice of placing the anti-CD3 moiety C-terminal to anti-WT1-HLA-A*02:01 moiety was based on structural modeling (**Supplementary Figure 2**). In the N-terminal placed anti-CD3, the anti-WT1-HLA-A*02:01 arms are pulled closed to the cell membrane by anti-CD3, potentially inducing steric clashed from the anti-WT1-HLA-A*02:01 and the Fc domains to the cell membrane. In addition, the distance between the two anti-WT1-HLA-A*02:01 is too close to achieve their parallel binding with HLA-A*0201 in its native orientation. As a result, the HLA-A*0201 have to re-orient to fit into an induced conformation. Hence the C-terminal placed anti-CD3 format is more relaxed and sterically favorable.

### The reduced distance between anti-WT1-HLA-A*02:01 Fab and anti-CD3 Fab in TED2 split results in enhanced cytotoxicity against tumor cells

The capability of WT1 TEDs to simultaneously bind WT1-HLA-A*02:01 and CD3 was confirmed *in vitro*. By performing a bio-layer interferometry concurrent binding assay where His-tagged CD3 was preloaded onto Penta-His sensors, we found that all three TEDs could be captured by CD3, and subsequently engage WT1-HLA-A*02:01 monomer (**Supplementary Figure 3A**). To examine the effects of the introduction of an additional anti-WT1-HLA-A*02:01 Fab on binding to WT1-HLA-A*02:01, we performed an Octet binding assay where streptavidin sensors were loaded with biotinylated WT1-HLA-A*02:01 monomer and subsequently dipped into different TEDs. Indeed, both TAA bivalent engaging formats - TED2 split and tandem - demonstrated enhanced binding avidity in comparison with TAA monovalent engaging TED (**Supplementary Figure 3B**).

We characterized binding of various WT1-HLA-A*02:01 positive tumor cell lines by all WT1 TED variants. Despite high HLA-A*02:01 density on the cell surface in the two cell lines, OVCAR3 and U266B1, negligible binding was observed for all three WT1 TED variants, indicating that flow cytometry was not sensitive enough to detect the low WT1-HLA-A*02:01 density (**Supplementary Figures 4**, **5**).

Next, we sought to determine whether WT1 TED2s could elicit enhanced cytotoxicity against tumor cells. In contrast to the WT1 TED that showed reduced cytotoxicity, WT1 TED2 split demonstrated significantly augmented cytotoxicity against two WT1-HLA-A*02:01^+^ tumor cell lines (**Figure 2A**; **Supplementary Figure 7**, **Supplementary Table 2**). WT1 TED2 tandem in which the second anti-WT1-HLA-A*02:01 Fab was fused in tandem with the original anti-WT1-HLA-A*02:01 arm showed lower activation compared to the parental WT1 TED, with a lower cytotoxicity profile. No cytotoxicity was observed against WT1-HLA-A*02:01^-^ tumor cell line K562, confirming that introduction of a second anti-WT1-HLA-A*02:01 Fab on the distal arm did not result in elevated nonspecific binding. This finding is in line with our hypothesis that the reduced distance between the newly introduced anti-WT1-HLA-A*02:01 Fab and anti-CD3 Fab, rather than the enhanced avidity, results in improved cytotoxicity.

As expected, the enhanced cytotoxicity of WT1 TED2 split against WT1-HLA-A*02:01^+^ tumor cell lines, was followed by elevated T cell activation by WT1 TED2 split (**Figure 2B**). In addition, WT1 TED2 split elicited higher levels of inflammatory cytokines, including interferon-γ (IFNγ), interleukin-2 (IL-2), tumor necrosis factor-α (TNFα), and interleukin-6 (IL-6) (**Figure 2C**).

To examine whether a monovalent anti-TAA Fab was sufficient for targeting high density receptors on tumor cells, we generated anti-EGFR (clone GA201)/anti-CD3 (clone UCHT1) TED (hereafter EGFR TED) to test its cytotoxicity against the OVCAR3 cell line. Both anti-TAA arms have single digit nM affinity with GA201 at 0.7 nM (internal data) and anti-WT1-HLA-A*02:01 clone ESK1 at 0.2 nM. High level EGFR expression was observed on OVCAR3 cells (**Supplementary Figure 5**). Consistent with the high receptor density, EGFR TED potently killed OVCAR3 cells at the same E:T ratio as for WT1 TED2 split (**Supplementary Figure 7**), indicating the original conformation between anti-TAA arm and anti-CD3 arm in EGFR TED is sufficient for IS formation in targeting high density TAAs.

**Figure 3:**
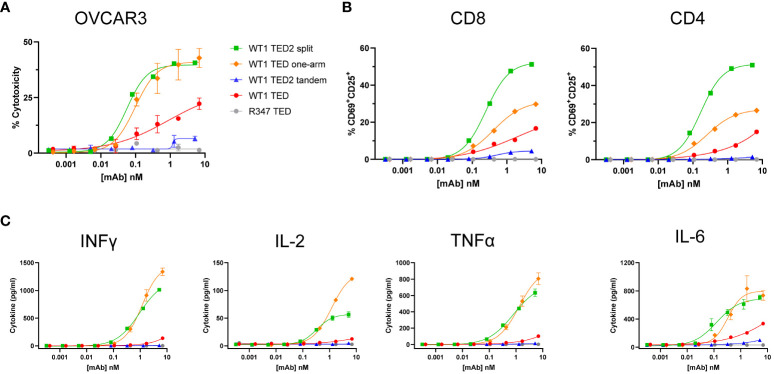
WT1 TED one-arm elicits potent cytotoxicity comparable to WT1 TED2 split against WT1-HLA-A*02:01+ tumor cells. **(A)** Cytotoxicity induced by WT1 TEDs against OVCAR3 cells. Primary T cells were incubated with target cells at E:T=5:1 for 24h and target cell cytotoxicity was assessed by FACS. **(B)** T cell activation induced by WT1 TEDs, evaluated by T cell surface CD69 and CD25 expression. **(C)** Inflammatory cytokine release elicited by WT1 TEDs against OVCAR3 cells. Cytotoxicity and cytokine concentrations were reported as the mean of two replicates, and error bar represents standard error of the mean. T cell activation was reported from one replicate. Representative data using primary T cells from one out of three donors were presented.

To further interrogate to what extent a second TAA arm contributed to the stabilized IS, we next evaluated the WT1 TED one-arm, only containing the distal anti-WT1-HLA-A*02:01 Fab N-terminal to the anti-CD3 (**Figures 1A, C**). Interestingly, WT1 TED one-arm exhibited similar biological activity as WT1 TED2 split, indicating that the proximity between the second anti-WT1-HLA-A*02:01 Fab and anti-CD3 Fab plays a more critical role compared to the enhanced avidity conferred by an additional TAA moiety on the distal arm (**Figure 3**; **Supplementary Table 2**).

### WT1 TED2 split elicits robust cytotoxicity against primary AML cells

**Figure 4:**
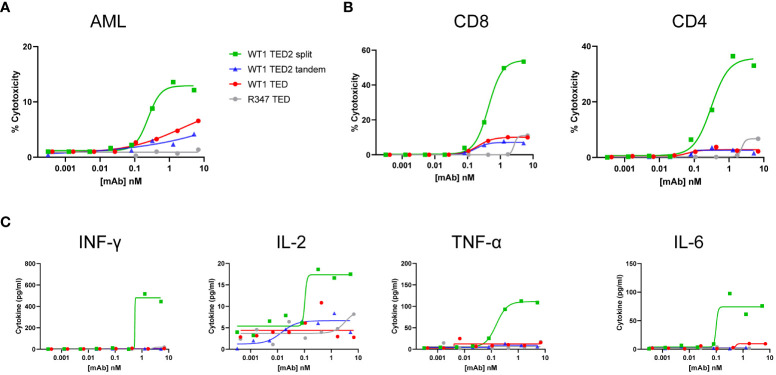
WT1 TED2 split elicits potent cytotoxicity against primary AML. **(A)** Cytotoxicity induced by WT1 TEDs against primary AML cells. Primary T cells were incubated with target cells at E:T=5:1 for 24h and target cell cytotoxicity was assessed by FACS. **(B)** T cell activation induced by WT1 TEDs, determined by T cell surface CD69 and CD25 expression. **(C)** Inflammatory cytokine release elicited by WT1 TEDs. Cytotoxicity and cytokine concentrations were reported as the mean of two replicates, and error bar represents standard error of the mean. T cell activation was reported from one replicate.

To further explore the utility of WT1 TED2 split to target WT1-HLA-A*02:01**^+^** tumors, we obtained WT1-HLA-A*02:01^+^ primary AML cells, given that primary AML cells have more physiological expression of WT1-HLA-A*02:01 than tumor cell lines. Out of two donors, one was identified as HLA-A*02:01^+^ (**Supplementary Figure 8A**). Indeed, WT1 TED2 split, and to a lesser extent WT1 TED and WT1 TED2 tandem, elicited robust cytotoxicity against primary AML cells (**Figure 4A**; **Supplementary Table 2**). WT1 TED2 split also induced substantial T cell activation and inflammatory cytokine release (**Figures 4B, C**). Negligible cytotoxicity was observed for WT1-HLA-A*02:01^-^ AML cells by WT1 TED2 (**Supplementary Figure 8B**).

Taken together, these data suggest that WT1 TED2 split can elicit potent cytotoxicity against tumor cells at physiological pMHC target density.

## Discussion

TCEs are very potent molecules, proven to be very efficient in re-targeting T cells and killing tumor cells, particularly when the surface antigens on the target cells are abundant (58). They have successfully been used to redirect T cells against blood and bone marrow cancers targeting CD19, CD20, BCMA or CD123 (2, 59–61). However, targeting of the attractive and elusive intracellular proteome as well as cell surface targets on solid tumors has proven to be challenging in the field of TCEs. In January of 2022, KIMMTRAK (Tebentafusp) by Immunocore was the first TCR therapeutic to gain FDA approval. KIMMTRAK targets a peptide from GP100 and was approved for the treatment of unresectable or metastatic uveal melanoma. KIMMTRAK is composed of single chain TCR alpha/beta variable domains fused to an anti-CD3 single-chain antibody and possesses low picomolar affinity towards pMHC (62, 63).

In addition to the approval of KIMMTRAK, promising data for TCEs targeting solid tumors has emerged from treatment of small cell lung cancer using DLL3 targeting TCEs AMG 757 (64) and HPN328 (65). Still, key challenges for TCEs are present for targeting solid tumors, including an immunosuppressive TME, poor penetration and limited density of the surface target (66).

In this study, we interrogated to what extent different arrangements of the TAA and the T cell targeting moiety in a TCE influence generation of a functional IS, in the context of low surface density antigens such as pMHC. To examine this, we generated and characterized a TCE targeting pMHC consisting of an anti-WT1-HLA-A*02:01/anti-CD3 based on the validated DuetMab modality (29–32, 43, 44).

By generation and evaluation of TEDs consisting of either one or two anti-WT1 placed at different locations, we found that the placement of the anti-TAA in relation to the anti-CD3 moiety is critical for generation of a functional IS and subsequent killing of the target cell. The added avidity of a second anti-TAA plays a minimal role as we show when comparing WT1 TED2 split and WT1 TED one-arm, clearly demonstrating the importance of the correct geometry versus added avidity when targeting low density targets. For a more physiological-relevant surface expression of the WT1-pMHC complexes, we targeted HLA-A*0201 positive primary AML cells and observed robust activation and subsequent cytokine release with the WT1 TED2 split variant. Interestingly, when targeting HLA-A*0201 positive cell lines or primary AML samples, the WT1 TED performed slightly better than the WT1 TED2 tandem. It appears that the simultaneous engagement of the N-terminal anti-WT1 moiety and the anti-CD3 moiety of WT1 TED2 tandem could contribute to further increased and suboptimal synapse distance relative to WT1 TED. In addition, having an additional TAA engaging moiety will likely change the geometry of the interaction between the target and the effector cell. For high density targets such as EGFR, the geometry is not as critical, as demonstrated via a conventional EGFR TED that kills the target cells efficiently. Altogether, this data indicates that it is indeed the IS stabilizing conformation formed by the proximity of the anti-WT1 Fab and anti-CD3 arm, and not the enhanced avidity resulting from the introduction of a second anti-WT1 Fab, that causes augmented cytotoxicity.

With the limited number of available surface targets specific to cancer cells, the ability to efficiently target cancer-specific intracellular proteins presented on pMHC has become more desirable and recently validated with KIMMTRAKs approval by FDA of the first bispecific peptide-HLA-directed CD3 T cell engager targeting gp100 in melanoma. All together highlighting opportunities for enhancing the selectivity of TCEs in the context of solid tumors via presentation of intracellular peptides by MHC. As described in this paper, careful considerations are key when going after low density targets such as pMHC.

Furthermore, we envision that using a similar design, TED2 can be engineered to target two distinct epitopes on the same TAA, or two different TAAs simultaneously, to enhance the tumor selectivity of T cell engagers and prevent tumor antigenic escape. Given the results of this study, careful placement of the effector recruiting arm is warranted and may also vary from target to target. We will be exploring *in vivo* models to assess the WT1 TED2 split format in upcoming studies.

While we have demonstrated that geometry plays a key role when targeting low density targets in the form of pMHC, there are several key factors that determine the efficacy of a TCE and its ability to form a functional IS. It is well established that the placement of the epitope on the target is important when designing TCEs. This becomes evident when targeting larger receptors, where the proximity to the membrane is important. Epitopes distal from the membrane cause suboptimal conditions for IS formation with poor lysis of the target cells. The epitope on the T cells also play an important role, for CD3-based engagers, the subunit of the complex that is targeted can have an impact on the efficacy of the TCE. In addition the affinity of the moieties also plays a key role, both on the anti-TAA and the anti-CD3 arm. The interplay between several factors determines the final efficacy as well as safety of a TCE.

Taken together, this study presents a promising and generalizable strategy to specifically target tumors with low density surface targets with full-length TCEs. We anticipate TED to be a potent T cell-redirecting therapy and the approach described herein to be broadly applicable toward enhancing efficacy against low density TAAs.

## Data availability statement

The original contributions presented in the study are included in the article/**Supplementary Material**. Further inquiries can be directed to the corresponding author.

## Ethics statement

Ethical approval was not required for the studies on humans in accordance with the local legislation and institutional requirements because only commercially available established cell lines were used.

## Author contributions

EW: Conceptualization, Investigation, Methodology, Project administration, Supervision, Writing – original draft, Writing – review & editing, Formal Analysis. BW: Conceptualization, Investigation, Writing – review & editing, Formal Analysis, Methodology, Writing – original draft. PP: Formal Analysis, Investigation, Writing – original draft, Writing – review & editing, Data curation. CY: Data curation, Formal Analysis, Writing – original draft, Writing – review & editing. PZ: Formal Analysis, Investigation, Writing – review & editing, Conceptualization, Supervision, Project administration. CZ: Data curation, Investigation, Writing – review & editing. YM: Investigation, Writing – original draft, Writing – review & editing, Conceptualization, Formal Analysis, Supervision. HZ: Data curation, Investigation, Writing – review & editing. PZ: Formal analysis, Writing - review and editing.

## Supplementary material

Supplementary Figure 1 Anti-CD3 is docked on TCR-CD3-HLA*0201 complex (PDB: 7PHR) (36) based on the crystal structure of αCD3-CD3 complex and AlphaFold modeling (38) (PDB: 1XIW) (33). Anti-WT1 is docked on the TCR-CD3-HLA*0201 complex based on the crystal structure of the anti-WT1-HLA-A2*0201 complex (PDB: 6RSY) (67). The inter-membrane distance between T cell and antigen-presenting cell (APC) and the distance between anti-CD3 and anti-WT1 epitopes are marked in the schematic.

Supplementary Figure 2 Modeling of WT1 TED2 split engaging an effector cell with a target cell. WT1 TED2 split, with the anti-WT1-HLA-A*02:01 N-terminal (Left) and C-terminal (right) are modeled on the TCR-CD3 complex (PDB: 6JXR) and HLA-A*0201 (PDB: 6RSY) based on the crystal structures of the Fab-antigen complex and AlphaFold modeling (PDB: 1XIW and PDB 4WUU).

Supplementary Figure 3 Binding of WT1 TEDs to soluble WT1-HLA-A*02:01 complex and CD3, measured with Octet. **(A)** Concurrent binding of WT1 TEDs to CD3 and WT1-HLA-A*02:01. Octet binding curves were generated by loading CD3 onto Penta-His sensors, followed by incubation with WT1 TEDs, and subsequently with WT1-HLA-A*02:01. EGFR TED was used as a negative control. **(B)** Increased avidity via an additional anti-WT1-HLA-A*02:01 Fab in WT1 TED2 split and tandem resulted in increased binding to WT1-HLA-A*02:01 compared to WT1 TED. EGFR TED was used as a negative control.

Supplementary Figure 4 HLA-A*02:01 expression on tumor cell lines. OVCAR3 and U266B1 cells were stained with anti-HLA-A2 antibody BB7.2. K562 cells were used as a negative control.

Supplementary Figure 5 Binding of WT1 TEDs on WT1-HLA-A*02:01^+^ tumor cells. Cellular binding of WT1 TEDs to WT1-HLA-A*02:01-expressing cell lines OVCAR3 and U266B1 are shown. EGFR TED was used as an example of a high density antigen.

Supplementary Figure 6 FACS gating strategy to assess TED mediated target cell cytotoxicity and T cell activation.

Supplementary Figure 7 EGFR TED elicits robust cytotoxicity against EGFR^+^ tumor cells. **(A)** EGFR TED induced cytotoxicity against OVCAR3 cells. Primary T cells were incubated with target cells at E:T=5:1 for 24h and target cell cytotoxicity was assessed by FACS. **(B)** EGFR TED induced T cell activation assessed by T cell surface CD69 and CD25 expression. Cytotoxicity was reported as the mean of two replicates, and error bar represents standard error of the mean. T cell activation was reported from one replicate. Representative data using primary T cells from one donor out of 3 were reported.

Supplementary Figure 8 WT1 TED2 split did not induce cytotoxicity against WT1-HLA-A*02:01^-^ primary AML. **(A)** HLA-A2 expression on AML cells from two donors, measured by binding of anti-HLA-A2 antibody BB7.2. AML from Donor 3 were used in . **(B)** Cytotoxicity elicited by WT1 TEDs against primary AML cells from Donor 2 in **(A)**. Primary T cells were incubated with target cells at E:T=5:1 for 24h and target cell cytotoxicity was assessed by FACS. Cytotoxicity was reported as the mean of two replicates, and error bar represents standard error of the mean.

Supplementary Table 1 Maximum distances between anti-WT1 and anti-CD3 paratopes in the different formats. As illustrated in **Figure 1C**, the maximum distances between paratopes are reached when the magenta-labeled points are coordinated into a straight line between anti-WT1 CDR H3 and anti-CD3 CDR H3.

Supplementary Table 2 Area under the curve calculations for **Figures 2A**, **3A** and **4C**. The total area under each curve (AUC) was calculated using GraphPad Prism.
