# Supplementary figures and images for: Conformation-selective rather than avidity-based binding to tumor associated antigen derived peptide-MHC enables targeting of WT1-pMHC low expressing cancer cells by anti-WT1-pMHC/CD3 T cell engagers
Source: Front Immunol. 2023 Nov 10;14:1275304. doi: 10.3389/fimmu.2023.1275304 (PMC10667733; doi:10.3389/fimmu.2023.1275304)

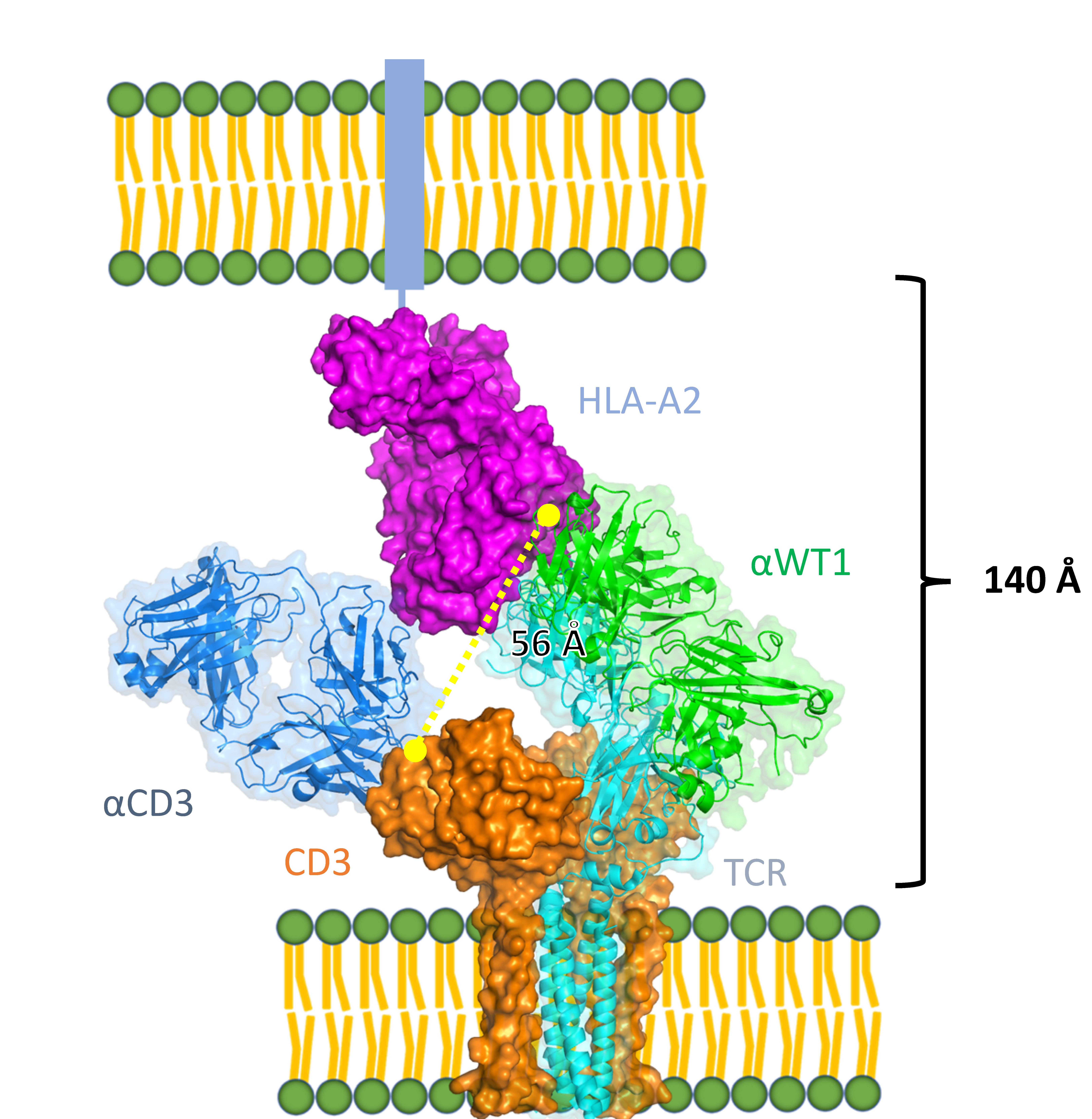

Supplement: Supplementary Figure 1 — Anti-CD3 is docked on TCR-CD3-HLA*0201 complex (PDB: 7PHR) (36) based on the crystal structure of αCD3-CD3 complex and AlphaFold modeling (38) (PDB: 1XIW) (33). Anti-WT1 is docked on the TCR-CD3-HLA*0201 complex based on the crystal structure of the anti-WT1-HLA-A2*0201 complex (PDB: 6RSY) (67). The inter-membrane distance between T cell and antigen-presenting cell (APC) and the distance between anti-CD3 and anti-WT1 epitopes are marked in the schematic. [file Image_1.jpg]

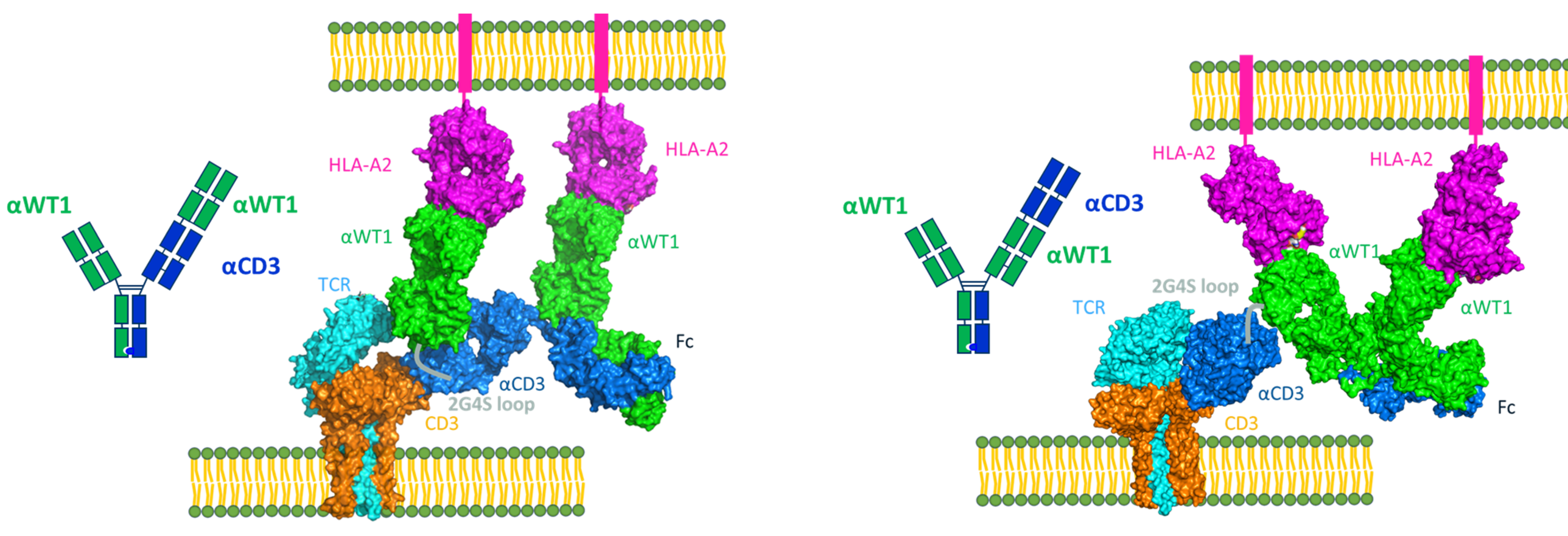

Supplement: Supplementary Figure 2 — Modeling of WT1 TED2 split engaging an effector cell with a target cell. WT1 TED2 split, with the anti-WT1-HLA-A*02:01 N-terminal (Left) and C-terminal (right) are modeled on the TCR-CD3 complex (PDB: 6JXR) and HLA-A*0201 (PDB: 6RSY) based on the crystal structures of the Fab-antigen complex and AlphaFold modeling (PDB: 1XIW and PDB 4WUU). [file Image_2.jpg]

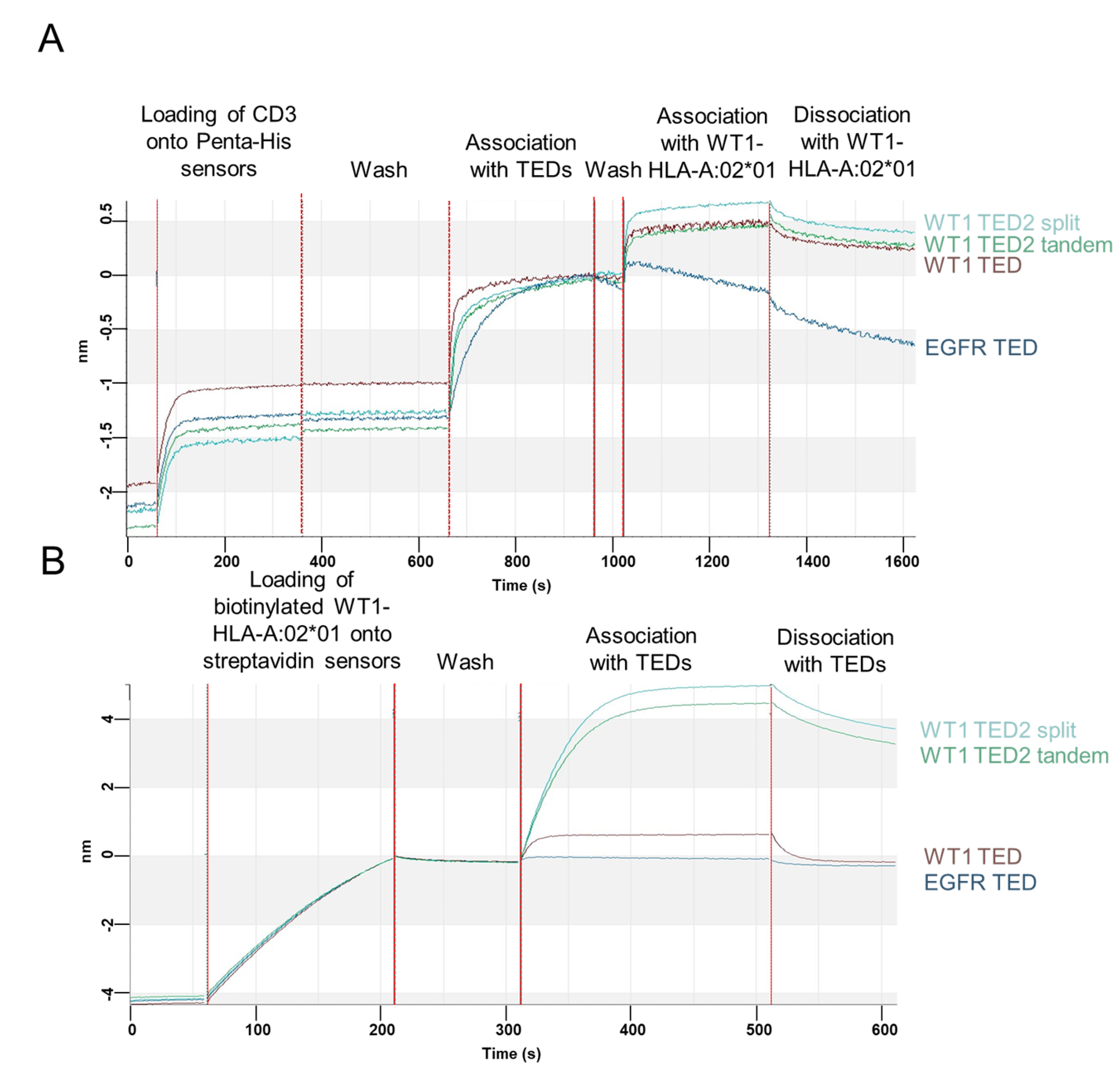

Supplement: Supplementary Figure 3 — Binding of WT1 TEDs to soluble WT1-HLA-A*02:01 complex and CD3, measured with Octet. (A) Concurrent binding of WT1 TEDs to CD3 and WT1-HLA-A*02:01. Octet binding curves were generated by loading CD3 onto Penta-His sensors, followed by incubation with WT1 TEDs, and subsequently with WT1-HLA-A*02:01. EGFR TED was used as a negative control. (B) Increased avidity via an additional anti-WT1-HLA-A*02:01 Fab in WT1 TED2 split and tandem resulted in increased binding to WT1-HLA-A*02:01 compared to WT1 TED. EGFR TED was used as a negative control. [file Image_3.jpg]

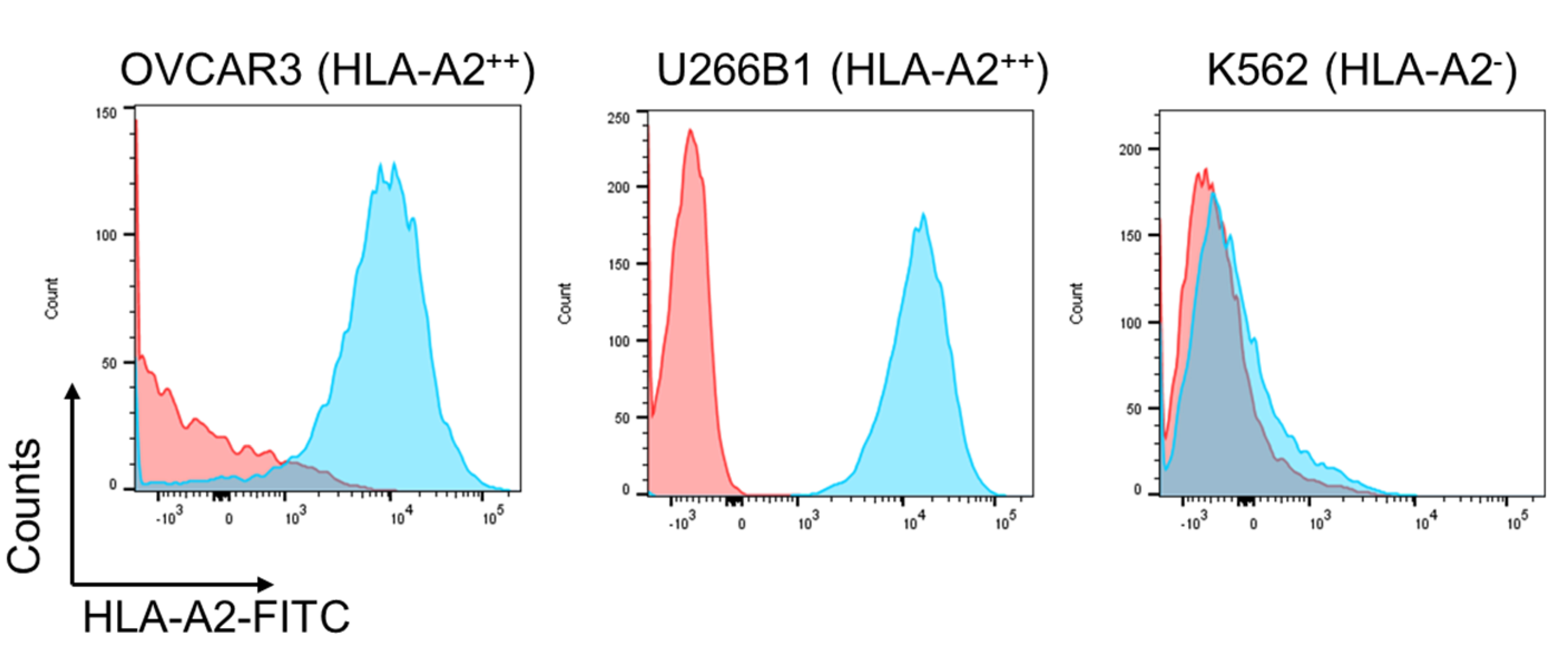

Supplement: Supplementary Figure 4 — HLA-A*02:01 expression on tumor cell lines. OVCAR3 and U266B1 cells were stained with anti-HLA-A2 antibody BB7.2. K562 cells were used as a negative control. [file Image_4.jpg]

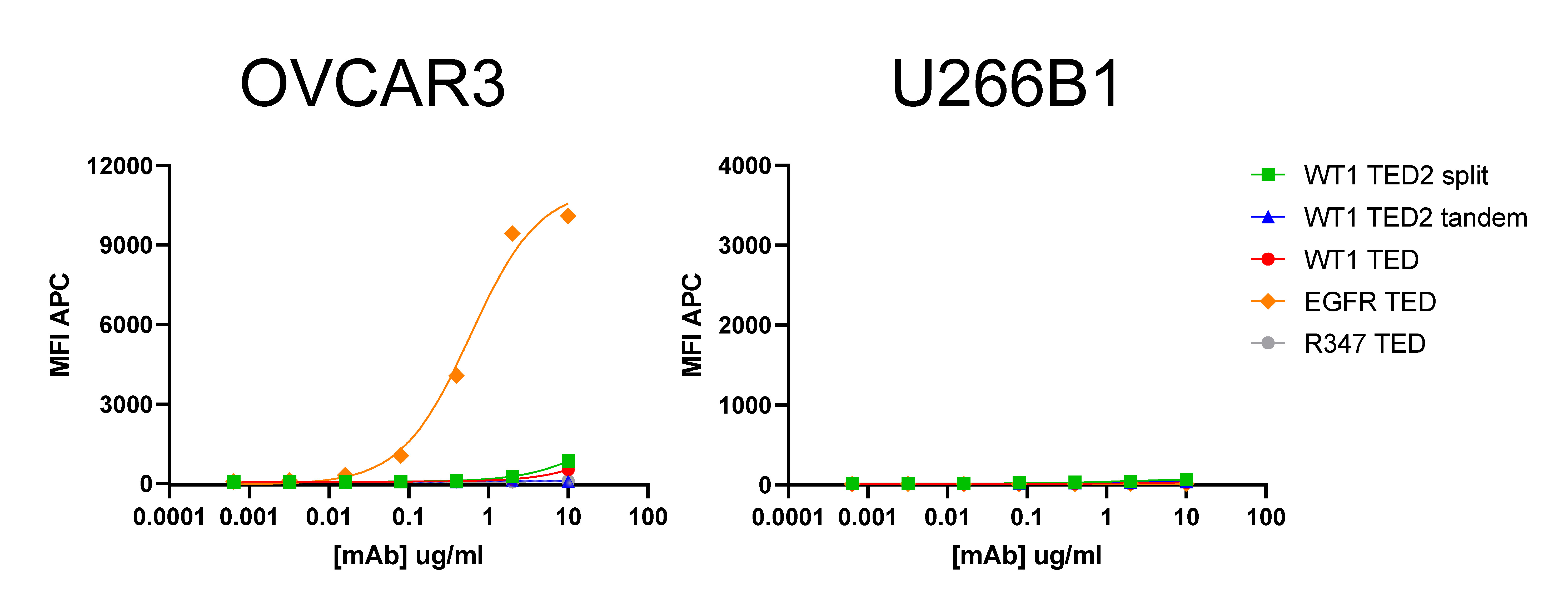

Supplement: Supplementary Figure 5 — Binding of WT1 TEDs on WT1-HLA-A*02:01+ tumor cells. Cellular binding of WT1 TEDs to WT1-HLA-A*02:01-expressing cell lines OVCAR3 and U266B1 are shown. EGFR TED was used as an example of a high density antigen. [file Image_5.jpg]

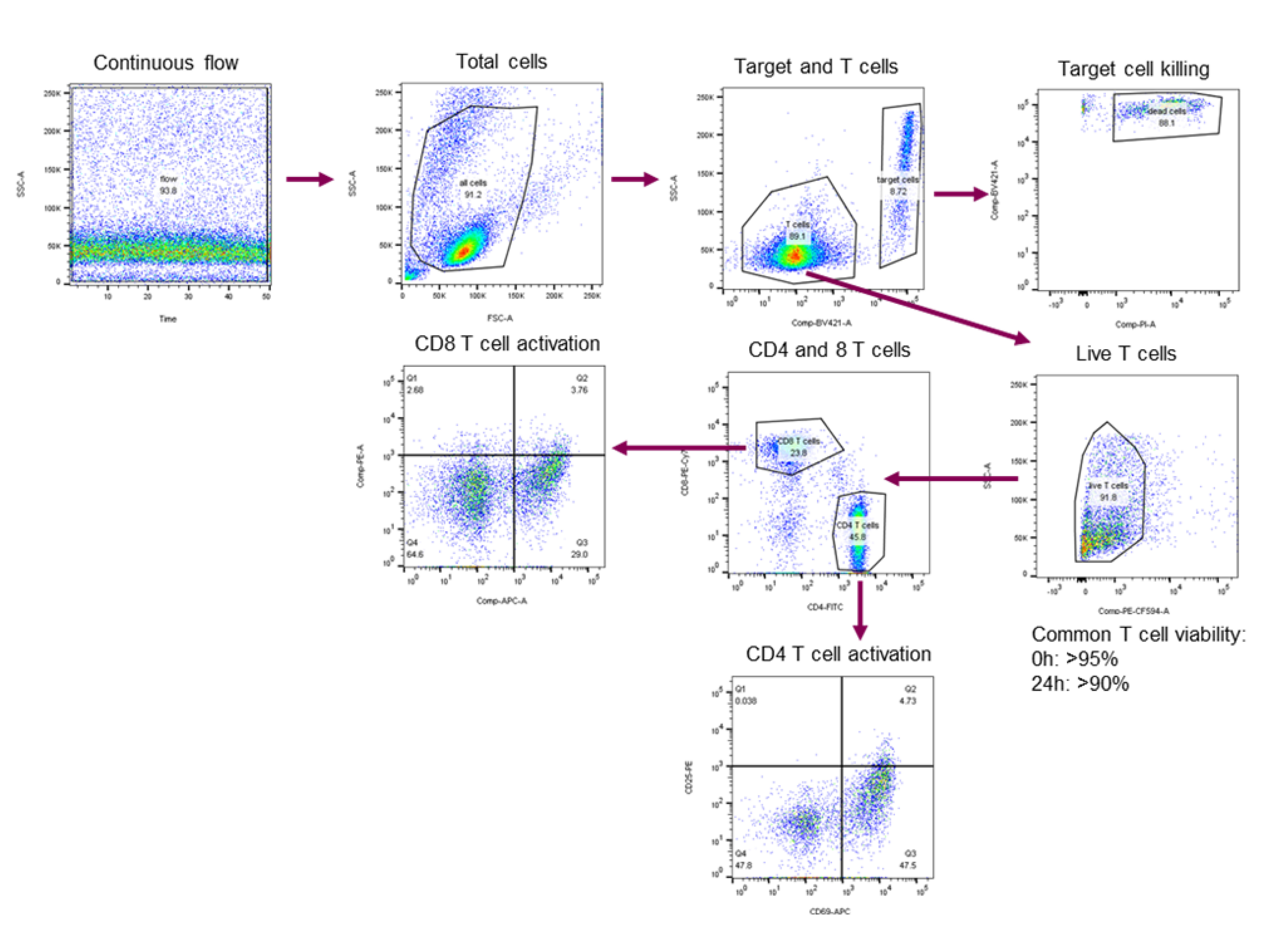

Supplement: Supplementary Figure 6 — FACS gating strategy to assess TED mediated target cell cytotoxicity and T cell activation. [file Image_6.jpg]

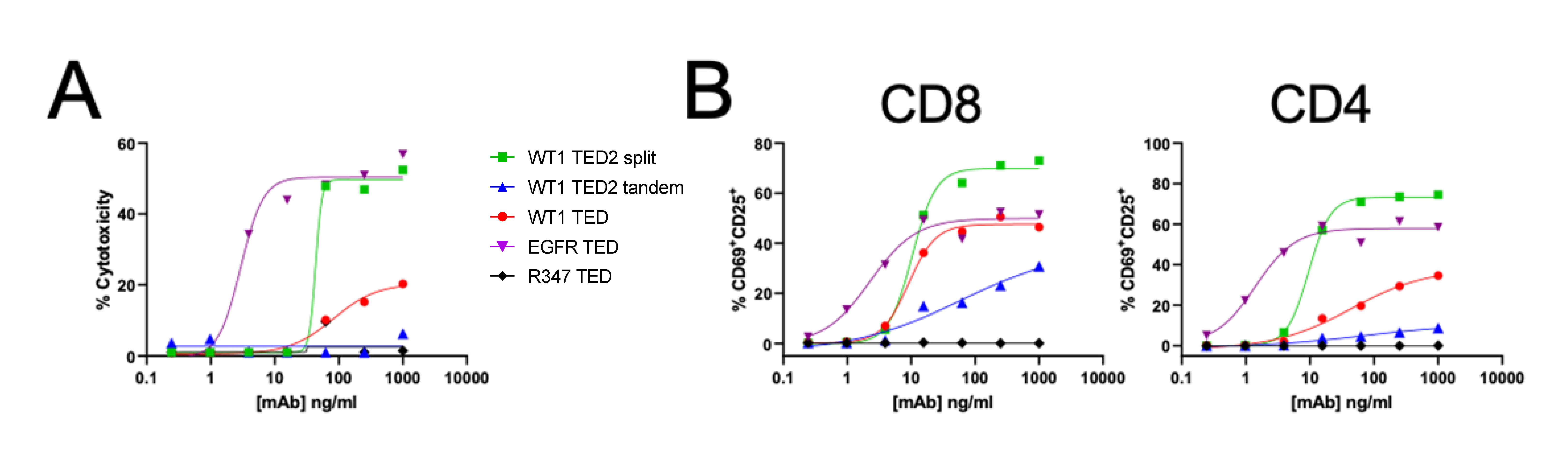

Supplement: Supplementary Figure 7 — EGFR TED elicits robust cytotoxicity against EGFR+ tumor cells. (A) EGFR TED induced cytotoxicity against OVCAR3 cells. Primary T cells were incubated with target cells at E:T=5:1 for 24h and target cell cytotoxicity was assessed by FACS. (B) EGFR TED induced T cell activation assessed by T cell surface CD69 and CD25 expression. Cytotoxicity was reported as the mean of two replicates, and error bar represents standard error of the mean. T cell activation was reported from one replicate. Representative data using primary T cells from one donor out of 3 were reported. [file Image_7.jpg]

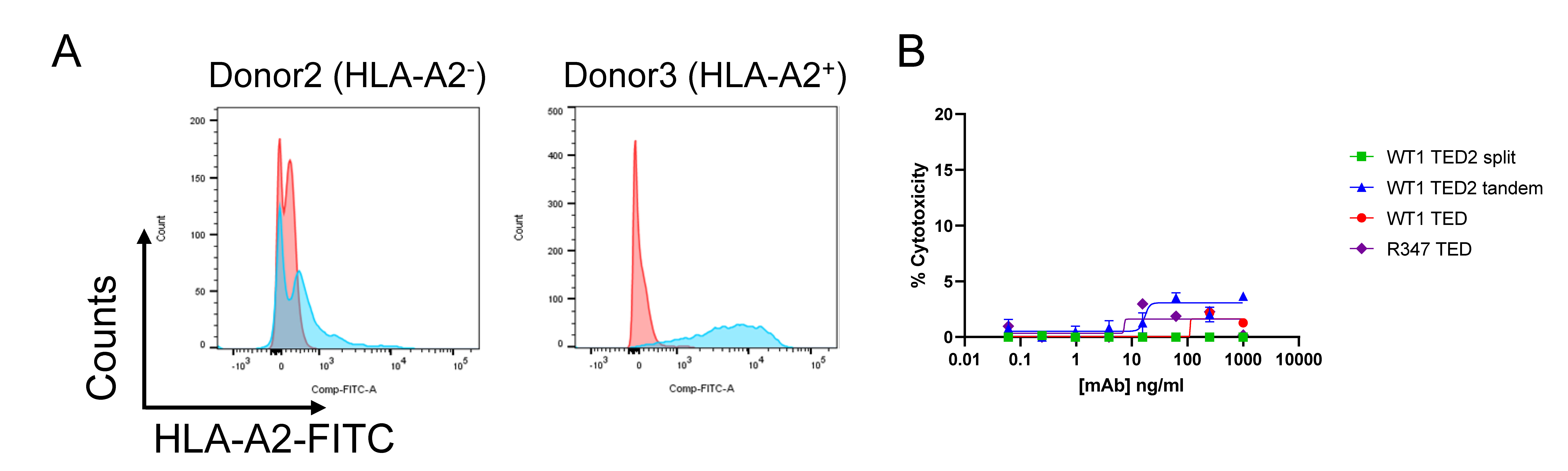

Supplement: Supplementary Figure 8 — WT1 TED2 split did not induce cytotoxicity against WT1-HLA-A*02:01- primary AML. (A) HLA-A2 expression on AML cells from two donors, measured by binding of anti-HLA-A2 antibody BB7.2. AML from Donor 3 were used in . (B) Cytotoxicity elicited by WT1 TEDs against primary AML cells from Donor 2 in (A). Primary T cells were incubated with target cells at E:T=5:1 for 24h and target cell cytotoxicity was assessed by FACS. Cytotoxicity was reported as the mean of two replicates, and error bar represents standard error of the mean. [file Image_8.jpg]

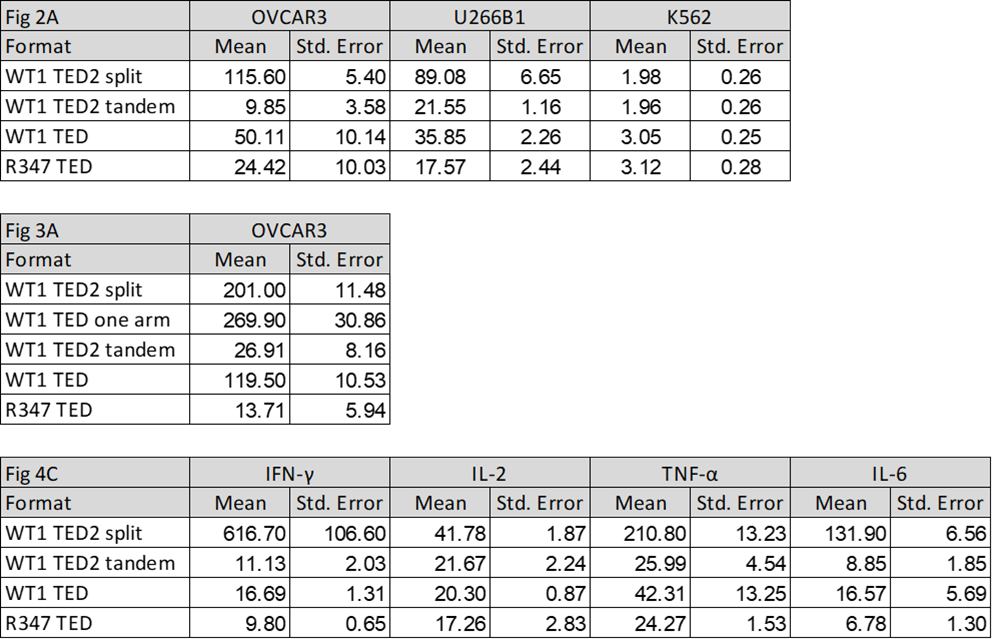


**Supplementary Table 2. Area under the curve calculations for figures 2A, 3A and 4C.**

Supplement: Supplementary Table 2 — Area under the curve calculations for Figures 2A , 3A and 4C . The total area under each curve (AUC) was calculated using GraphPad Prism. [file Table_2.docx]
