# Supplementary material for: Conformation-selective rather than avidity-based binding to tumor associated antigen derived peptide-MHC enables targeting of WT1-pMHC low expressing cancer cells by anti-WT1-pMHC/CD3 T cell engagers
Source: Front Immunol. 2023 Nov 10;14:1275304. doi: 10.3389/fimmu.2023.1275304 (PMC10667733; doi:10.3389/fimmu.2023.1275304)
Supplement: Supplementary Table 1 — Maximum distances between anti-WT1 and anti-CD3 paratopes in the different formats. As illustrated in Figure 1C , the maximum distances between paratopes are reached when the magenta-labeled points are coordinated into a straight line between anti-WT1 CDR H3 and anti-CD3 CDR H3. [file Table_1.docx]

**Supplementary Table 1. Maximum distances between anti-WT1 and anti-CD3 paratopes in the different formats.**
